# Seasonal connectivity of microbes and carbohydrates between ocean, atmosphere, and cryosphere in Kongsfjorden (Svalbard, Arctic Ocean)

**DOI:** 10.1093/ismeco/ycag212

**Published:** 2026-07-19

**Authors:** Matthias Wietz, Manuela van Pinxteren, Heike M Freese, Cathrin Spröer, Sebastian Zeppenfeld

**Affiliations:** Deep-Sea Ecology and Technology, Alfred Wegener Institute Helmholtz Centre for Polar and Marine Research, 27570 Bremerhaven, Germany; Max Planck Institute for Marine Microbiology, 28359 Bremen, Germany; Institute for Chemistry and Biology of the Marine Environment, University of Oldenburg, 26129 Oldenburg, Germany; Atmospheric Chemistry Department, Leibniz Institute for Tropospheric Research, 04318 Leipzig, Germany; Department of Bioinformatics and Databases, Leibniz Institute DSMZ − German Collection for Microorganisms and Cell Cultures, 38124 Braunschweig, Germany; Department of Bioinformatics and Databases, Leibniz Institute DSMZ − German Collection for Microorganisms and Cell Cultures, 38124 Braunschweig, Germany; Atmospheric Chemistry Department, Leibniz Institute for Tropospheric Research, 04318 Leipzig, Germany

**Keywords:** Arctic, prokaryotes, microeukaryotes, sea-air connectivity, chemistry, meteorology, snow, seasonal sourcing, metabarcoding, genome-sequenced model isolates

## Abstract

The coupling between ocean and atmosphere across the strong seasonal gradients in the Arctic is poorly understood. Here, we explored the microbial and glycobiological connectivity between the surface microlayer, the underlying seawater, snow, and aerosol particles in Kongsfjorden (Svalbard, 79°N) during autumn and spring. The marked overlap between marine and atmospheric microbiomes illustrates considerable sea-air transfer, linked to seasonally distinct environmental communities. For instance, *Polaribacter* and *Formosa* were aerosolized during the spring bloom, compared to *Colwellia* in autumn. Air-mass histories and microbial source tracking revealed a greater marine contribution in autumn, whereas spring aerosols were shaped by stronger winds and the cryosphere. Aerosol particles nonetheless contained numerous unique taxa, including Actinomycetes and Planctomycetia. Linking bacterial, microeukaryotic, carbohydrate, and meteorological dynamics established an overarching perspective across seasons and habitats, identifying five distinct ecosystem states. Genome-sequenced bacterial model isolates, representing key environmental populations, encode adaptive traits such as carotenoid and ectoine biosynthesis, linked to niche specialization and atmospheric transfer. Time-series records from the nearby Fram Strait revealed that many aerosolized taxa are core microbiome components; shaping ecology and biogeochemistry across the wider Arctic.

## Graphical abstract

**Figure:**
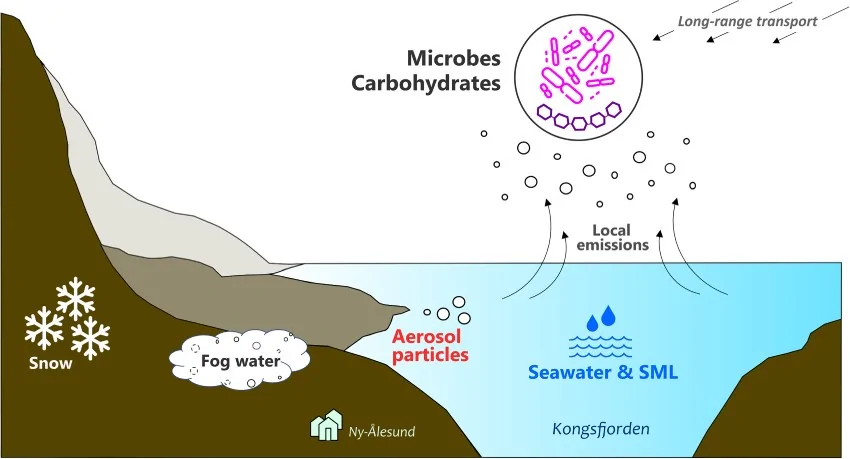

## Introduction

Around the Svalbard archipelago in the high Arctic, microbial and biogeochemical regimes show distinct seasonality [1–3]. Comparable patterns occur in the Arctic atmosphere [4–6]. However, the seasonal connectivity between aquatic and atmospheric realms—integrating microbiology, chemistry, and ecology—remains underexplored.

Aerosol particles are key connectors between seawater and atmosphere, mediating the transfer of diverse microbial and chemical components [7–9]. The aerosolization of waterborne components mainly occurs through bubble bursting at the surface microlayer (SML). Organic matter and cells are often enriched in the SML compared to the underlying water, coincident with specific chemical signatures and microbial adaptations [10–13]. The aerosolization of microbes aligns with their genetic and phenotypic traits [14, 15], and can vary by season and the environmental origin; for instance terrestrial, marine, or cryosphere [16–18]. Meteorological conditions play a key role in this context [19–21], including atmospheric dispersal over considerable distances [22–25].

The strong seasonality in the Arctic—featuring seasonally distinct microbial taxa, genetic functions, and substrate regimes—likely coincides with marked contrasts in ocean–atmosphere connectivity. Arctic aerosol particles mostly originate from local marine sources, especially the SML, plus long-range advection and terrestrial input [4, 26, 27]. These dynamics align with the phytoplankton bloom stage and associated production of carbohydrates, amino acids, and volatiles [28]. For instance, D- and L-amino acids have been suggested as “aerosol indicators” for early- and post-bloom respectively; together with the enrichment of specific microbes along the bloom succession [27, 29]. Aerosolized compounds might undergo microbial transformation in the air [20, 30], supported by laboratory experiments [31].

Here, we explore microbial communities and carbohydrates in the SML, the underlying seawater, and aerosol particles during autumn 2021 and spring 2022 in Kongsfjorden, Svalbard (79°N). These times mark the late and early phytoplankton bloom, featuring distinct microbial genera and environmental conditions [32–35]. Kongsfjorden and adjacent waters have been extensively studied regarding microbial, biogeochemical, and environmental dynamics across seasons and years [36–42]. We hypothesize that seasonal changes in marine-ecological and meteorological conditions shape both the waterborne and atmospheric microbiome; from autotrophic phytoplankton to heterotrophic bacteria. Carbohydrates are a key component of these dynamics [43, 44]. Accordingly, a study conducted alongside the present work has revealed pronounced seasonality in marine carbohydrate budgets and their atmospheric transfer [45]. We demonstrate marked sea–air connectivity of prokaryotes, microeukaryotes and carbohydrates, supported by genomic analyses of model isolates and seasonal microbiome records from the nearby Fram Strait [33, 34]. Given the exceptional warming of the Arctic [25] and the projected impact of climate change on the airborne microbiome [46], the sources and sinks of microbes and molecules are key aspects for understanding the microbial loop.

## Materials and methods

**Figure 1:**
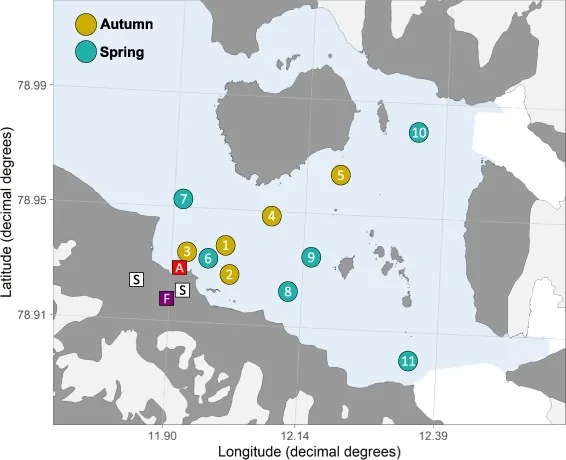
Sample locations across Kongsfjorden, indicating ULW and SML samplings as colored circles (yellow – autumn, green – spring). Aerosol particle (A), snow (S), and fog water (F) samplings are indicated by red, white and purple boxes, respectively. See Supplementary Table S1 for details.

**Seasonal sampling.** Samples were obtained during the BELUGA campaign at the AWIPEV research station (Kongsfjorden, Ny-Ålesund, Svalbard) in September–October 2021 and April–May 2022 (Fig. 1, Supplementary Table S1). The sampling procedure is detailed in a parallel atmospheric study [45]. Briefly, aerosol particles were collected over 4- to 7-day intervals on 47 mm polycarbonate membranes between September 25–October 30 (five samples) and April 30–May 14 (three samples) at the Old Pier next to the shoreline. Flow rates were 5–10 L min^−1^, with total air volumes of 44–82 m^3^. During aerosol collection, the SML and underlying seawater (ULW) were sampled from a motorized dinghy (five stations in autumn, six in spring). SML samples were collected by immersing a glass plate (surface area 2000 cm^2^) vertically in the seawater, withdrawing the plate at 15 cm s^−1^, and transferring the attached film into a clean plastic bottle using a funnel and a Teflon wiper. The corresponding ULW was sampled from 1 m depth with a low-density polyethylene bottle on a telescopic rod. A subsample of the same ULW/SML was used for carbohydrate quantification (see below). Spring snow was sampled ~500 m inland using ethanol-cleaned shovels and plastic containers. Each 1 L SML, ULW, and melted snow were sequentially filtered (47 mm polycarbonate membranes with 3 and 0.2 μm pore size) using mild vacuum (200 mbar) to distinguish microbial size classes: planktonic bacteria/small protists (FL) and attached bacteria/larger eukaryotes (PA). All filters were immediately frozen at −20°C until DNA extraction and metabarcoding of 16S and 18S rRNA genes. Fog water was sampled on the roof of the Gruvebadet Observatory as detailed in [47]. Briefly, fog droplets were captured on 508 μm diameter Teflon strands and transferred into clean polyethylene bottles; at a flow rate of 5.3 m^3^ min^−1^ and a 50% lower droplet cut-off diameter of ~3.5 μm.

**Microbial metabarcoding.** Microbial communities were analyzed in line with the FRAM Observatory [32]. Briefly, 16S and 18S rRNA gene fragments were MiSeq-sequenced using primers 515F-926R and 528iF-964iR [48, 49], processed into amplicon sequence variants using DADA2 v1.14.1 [50], and taxonomically classified using Silva v138.2 and PR^2^ v5.1.1 [51, 52]. Singleton, mitochondria, chloroplast, and metazoan reads were discarded. To account for potential contaminants, counts from PCR negative controls were subtracted from the raw abundance tables. Sequence results were further carefully inspected manually, and potential contaminants removed by comparison with literature data.

**Bacterial isolation, genome sequencing, and comparative genomics.** Bacterial strains were isolated from enrichment cultures in minimal seawater medium [53] with arabinogalactan, fucoidan, or alpha-mannan as sole carbon sources. Strains were pure-cultured on minimal seawater agar containing 0.4% glucose and 0.2% casamino acids (w/v). 16S rRNA gene fragments were Sanger-sequenced using primers 27F-907RM at LGC Genomics (Berlin, Germany). Using Geneious Prime, we identified five isolates that match Kongsfjorden ASVs at 100% rRNA gene identity (Supplementary Table S2). These strains were whole-genome sequenced on Sequel IIe and Revio systems (Pacific Biosciences, Menlo Park, CA) taking one 30 h movie per SMRT cell. For this, genomic DNA was extracted using the MasterPure Gram Positive DNA Purification Kit (Epicentre Biotechnologies, Germany) according to the manufacturer’s instructions. Samples were pooled equimolar. A template library was prepared using the SMRTbell kit 3.0, following the protocol “Preparing whole genome and metagenome libraries'' to ensure optimal primer annealing and polymerase binding to purified SMRTbell template. Loading was done using the calculator in SMRT Link v13.1. Long-read assemblies were performed with the ``Microbial Genome Analysis” protocol in SMRT Link v13.1. For potentially mixed cultures (NYA28a, NYA30) the dominant strain was retrieved though an additional assembly with Flye v2.9 [54]. Assemblies were circularized, the start point adjusted (*dnaA* for chromosomes), and corrected via mapping of PacBio reads using Minimap2 v2.28 [55] and variant detection using VarScan v2.28 [56]. Closed genomes were annotated using Bakta v1.11.3 [57]. CAZymes, biosynthetic gene clusters, and metabolic genes were annotated using dbCAN3, antiSMASH v8.0, KofamKOALA, and KEGG Mapper [58–61].

**Carbohydrate quantification.** To quantify carbohydrates in particulate (pCCHO, >0.2 μm) and dissolved (dCCHO/dFCHO, <0.2 μm) phases, seawater samples were analyzed according to [62, 63] via high-performance anion-exchange chromatography with pulsed amperometric detection (HPAEC-PAD), utilizing a Dionex CarboPac PA20 analytical column (3×150 mm) and a PA20 guard column (3×30 mm). The monosaccharides fucose, rhamnose, arabinose, galactose, glucose, xylose, mannose, fructose, galactosamine, glucosamine, muramic acid, galacturonic acid, and glucuronic acid were identified by their retention times. dFCHO represent identifiable free monosaccharides, whereas dCCHO and pCCHO include those released following acid hydrolysis (0.8 M HCl, 100°C, 20 h). Total combined carbohydrates (CCHO) represent the sum of dCCHO and pCCHO, and CHO the sum of CCHO and dFCHO. Carbohydrates in aerosol particles were measured from aqueous extracts with (CCHO_aer_) or without (dFCHO_aer_) acid hydrolysis.

**Meteorological conditions and air mass histories.** Data from the AWIPEV atmospheric observatory (https://doi.org/10.1594/PANGAEA.914979) were used to calculate wind speed, wind direction, air temperature, and air pressure across the aerosol sampling. Furthermore, 48-h back-trajectories (generated hourly at arrival altitudes of 50, 250, and 1000 m) were computed using the NOAA HYSPLIT model [64] based on GDAS1 meteorological fields (Global Data Assimilation System; 1° latitude/longitude; 3-hourly). Sea-ice concentrations were retrieved from ERDDAP (https://opendap.co-ops.nos.noaa.gov/erddap/index.html). To analyze air mass histories in relation to surface types, back-trajectories and sea-ice maps were integrated to calculate the relative residence time of air masses over distinct areas before sampling. Surface types were classified into open ocean (sea-ice concentration < 15%), marginal ice zone (sea-ice concentration 15%–80%), pack ice (sea-ice concentration 80%–100%), and land.

**Statistical evaluation.** Data were analyzed in RStudio using R v4.5.1 and packages *tidyverse*, *gtools*, *fuzzyjoin*, *cowplot*, *PlotSvalbard*, *ggOceanMaps*, *ggspatial*, *solartime, oce*, *ocedata*, *ncdf4*, *openair*, *cmocean*, *maps*, *mapdata*, *scales*, *lubridate*, *sp*, *rgdal*, *raster*, and *RColorBrewer* for data processing and plotting [65–84]; *ampvis2* for ordination and heatmaps [85], *rstatix* for Wilcoxon and Dunn’s tests [86], *UpSetR* for presense-absence analyses [87], *FEAST* for microbial source tracking [88], and *iNEXT* for alpha-diversity metrics [89]. PERMANOVAs were done using Bray-Curtis dissimilarities on Hellinger-transformed relative abundances in *vegan* [90]. Partial least squares regression (sPLS) using packages *mixOmics*, *amap*, and *ape* [91–93] identified associations between Hellinger-transformed abundances and environmental variables. ENVO terms were assigned in accordance with Lang-Yona and colleagues [94]. Figures were arranged and beautified using Inkscape. All bioinformatic code and data files are available under https://github.com/matthiaswietz/BelugaMicrobes.

**Data availability.** Amplicon sequences are available under ENA accession number PRJEB91661; deposited via the German Federation for Biological Data in compliance with MIxS standards. Closed bacterial genomes are available under ENA accession number PRJEB91655. Carbohydrate and ion concentrations in ULW and SML are available under https://doi.org/10.1594/PANGAEA.982606. Carbohydrate concentrations in aerosol particles are available under https://doi.org/10.1594/PANGAEA.982703.

## Results and discussion

Environmental samples were collected in autumn 2021 and spring 2022 in Kongsfjorden, Svalbard (79°N 11°E; Fig. 1, Supplementary Tables S1–S3). Aerosol particles were collected over 4- to 7-day intervals between September 25–October 30 (five samples) and April 30–May 14 (three samples) next to the shoreline. During these periods, the SML and the ULW were sampled at five and six stations, respectively. Spring snow was sampled at two sites ~500 m inland. SML, ULW and melted snow were sequentially filtered to distinguish planktonic bacteria/small protists (FL) and attached bacteria/larger eukaryotes (PA). Their composition was analyzed through 16S and 18S rRNA amplicon sequencing. Microbial dynamics were contextualized with carbohydrate concentrations measured from the same set of samples, genomic traits of bacterial isolates, meteorological conditions, and year-round microbiome records from the nearby Fram Strait [33, 34].

### Overall microbiome structure

**Figure 2:**
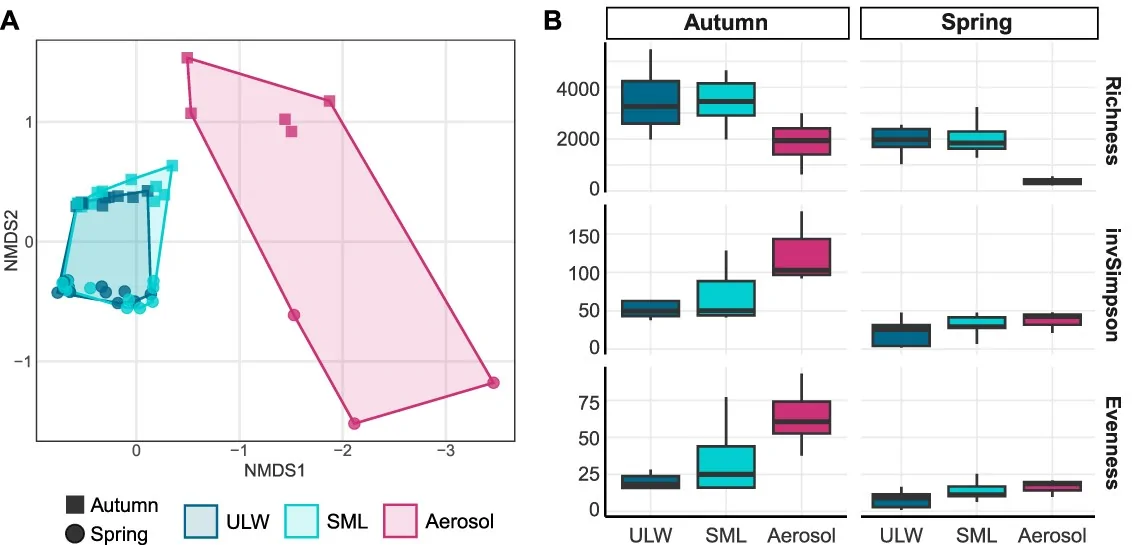
Seasonal microbial dynamics. Non-metric multidimensional scaling of Bray-Curtis dissimilarities (A; stress value 0.07) and alpha-diversity (B) of bacterial communities in the SML, the ULW, and aerosol particles. Significance was demonstrated by PERMANOVA (*P* < .001) and Kruskal-Wallis tests (*P* < .05), respectively. See Supplementary Fig. S1 for the corresponding seasonal dynamics of microeukaryotes.

**Figure 3:**
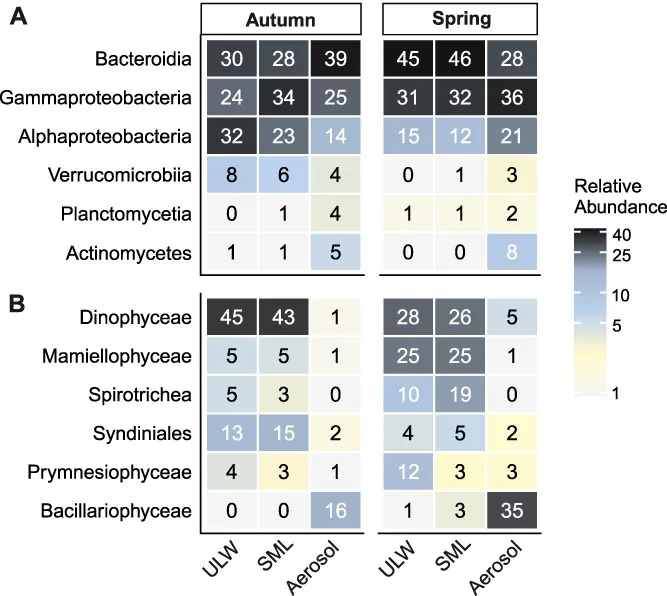
Seasonal microbiome composition. Average relative abundances of the major bacterial (A) and microeukaryotic (B) classes in SML, ULW, and aerosol particles in autumn and spring.

SML und ULW microbiomes were largely congruent and shared a clear seasonal trajectory, both for bacteria and microeukaryotes (PERMANOVA; *R^2^* ≈ 0.2, *P* < .001; Fig. 2, Supplementary Fig. S1, Supplementary Table S2). Bacterial diversity and evenness were significantly higher in autumn (Supplementary Table S2; Dunn’s test, adjusted *P* < .01), linked to higher abundances of Alphaproteobacteria and Verrucomicrobiia while Bacteroidia peaked in spring (Fig. 3). Aerosol microbiomes showed comparable seasonality (PERMANOVA; *R*^2^ = 0.2, *P* < .05). The higher diversity and evenness in aerosol particles compared to ULW and SML (Fig. 2, Supplementary Table S2; Dunn’s test, adjusted *P* < .03) reflected higher abundances of Actinomycetes and Planctomycetia (Fig. 3). Despite these differences, numerous ASVs were shared between ocean and atmosphere (see below).

Eukaryotic diversity also peaked in autumn, but with consistent values among ULW, SML, and aerosol particles (Supplementary Fig. S1). The predominance of Dinophyceae and Syndiniales in autumn reflects mixo- and heterotrophy in the late productive season. Photosynthetic microalgae (prymnesiophytes and diatoms), prasinophytes (*Mamiellophyceae*), as well as ciliates (*Spirotrichea*) predominated in spring (Fig. 3). Overall, autumn and spring dynamics are consistent with reported seasonal patterns around Svalbard [32, 95–97].

We now focus on bacterial aerosolization and environmental sourcing, before returning to an ecosystem-scale analysis below. Eukaryotes—with larger cell sizes but lower cell numbers than bacteria—were excluded from detailed aerosolization analyses, as they are presumably aerosolized more randomly, resulting in greater stochasticity as indicated by the larger spread in community ordination (Supplementary Fig. S1).

### Bacterial distribution and traits across environments

**Figure 4:**
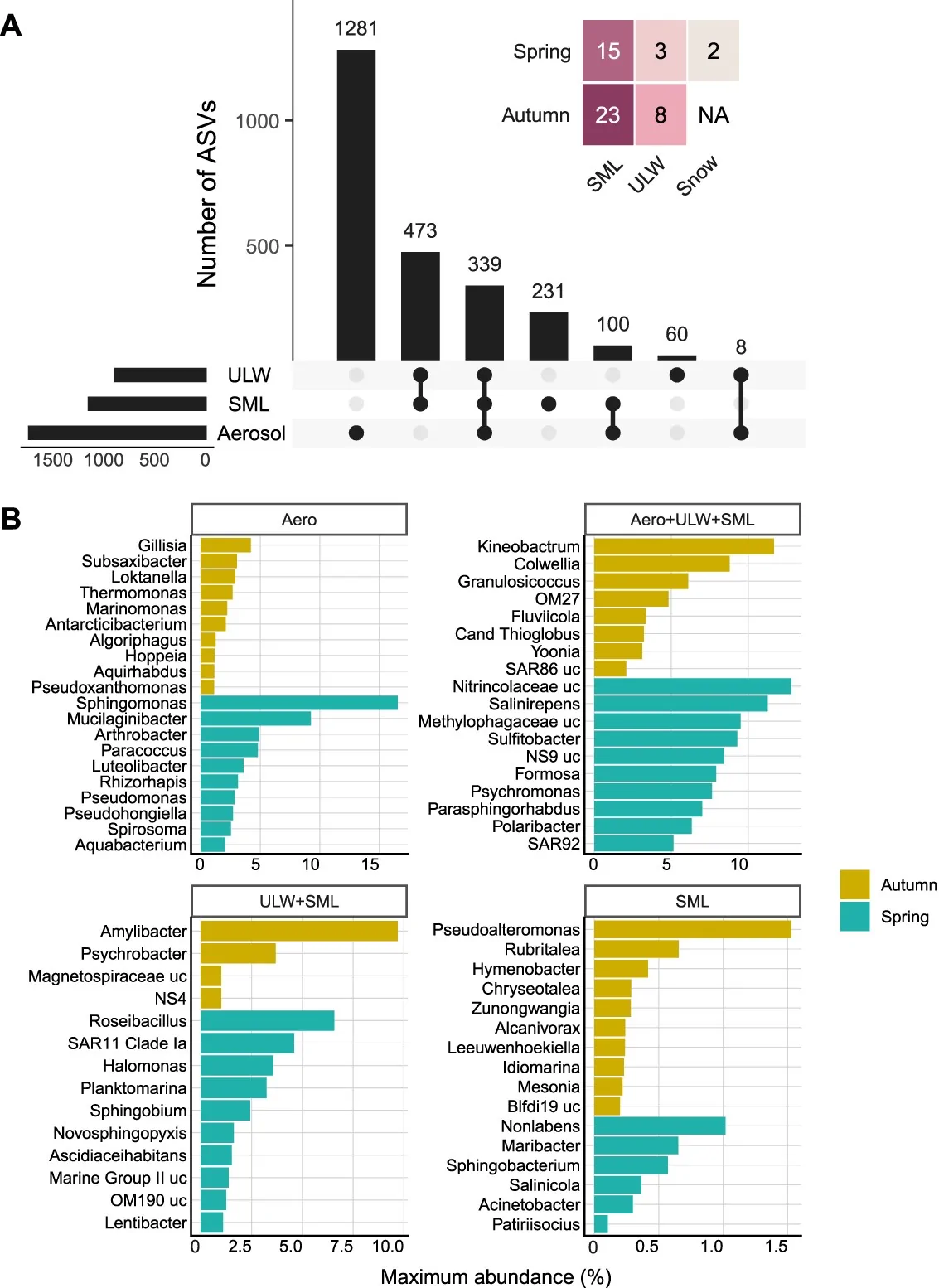
Bacterial composition across environments and seasons. (A) Shared and unique ASVs in SML, ULW, and aerosol particles, determined by presence-absence analysis. The upset plot illustrates the number of ASVs shared across all combinations of environments. The upper-right insert shows percent overlap between ULW, SML, and snow microbiomes with those in aerosol particles, as determined by source tracking. No snow was sampled in autumn; hence no overlap could be calculated. (B) Predominant genera per environment based on the 50 most abundant ASVs, showing the maximum abundance in their peak season. uc: unclassified (only assigned to family level).

We explored the distribution and connectivity of bacteria in seawater and atmosphere. Aerosol particles harbored ~1300 unique bacterial ASVs, which however only constituted ~8% of the aerosol particle community (ie, many but rare taxa in line with alpha-diversity; Fig. 2). The prevalence of Actinomycetes (Fig. 3) corresponded to diverse taxa including *Arthrobacter*, *Nocardioides*, and *Mycobacterium* (Fig. 4). Microbial source tracking—ie, calculating community overlap between environments—showed that aerosol microbiomes were more similar to SML than ULW microbiomes (Fig. 4), highlighting the SML’s role in sea-air connectivity [98]. Still, the majority of aerosolized ASVs occurred in both SML and ULW, constituting an average abundance of ~58%. Shared ocean–atmosphere taxa included *Polaribacter*, *Formosa* and *Methylophagaceae* (Fig. 4)—key drivers of carbohydrate and methanol cycling during phytoplankton blooms [43, 99]. *Polaribacter* was similarly abundant in aerosol particles during a preceding spring bloom in Kongsfjorden [29], indicating frequent aerosolization during high primary production. *Formosa* correlated with ice-nucleating particle concentrations near Greenland while being metabolically active [27], linking bacterial aerosolization to carbohydrate transformations.

We calculated three indices expressing the relative abundance ratio of each ASV between environments: the Aerosolization Index (aerosol particles compared to ULW + SML), the SML Index (SML compared to ULW), and the Attachment Index (PA compared to FL). SML and Attachment indices peaked in autumn and were positively correlated (Spearman’s ρ = 0.63; *P* < .001), illustrating two key patterns: more particles in the SML, reflecting the known enrichment of organic matter [100], and more PA bacteria in autumn. Accordingly, SML taxa predominated in the PA fraction (~64%), compared to ULW taxa in the FL fraction (~63%). The positive correlation between Aerosolization and Attachment indices (Spearman’s ρ = 0.2; *P* < .001) underlines that PA bacteria are favorably aerosolized. Consequently, the mostly free-living taxa SAR11 and *Amylibacter* [101, 102] were absent in aerosol particles. Nonetheless, their presence in autumn fog water collected hundreds of meters inland (Supplementary Fig. S2) indicates atmospheric niches for bacteria with reportedly little aerosolization [14]. The fog water furthermore comprised terrestrial taxa like *Pseudomonas* and *Arcicella*, indicating sourcing from soils [103].

Adaptations to salinity and UV stress are essential for survival in the SML [104, 105], and our genome-sequenced isolates illustrate the underlying traits. *Salinicola* sp. NYA28a and *Halomonas* sp. NYA30—matching asv647 and asv310 and hence representing relevant environmental populations—predominate in the SML (Fig. 4, Supplementary Fig. S3). Both isolates belong to the salinity-tolerant *Halomonadaceae* family and encode identical ectoine biosynthesis clusters (Supplementary Table S2), reflecting adaptation to the rapid osmotic shifts in the SML [104]. NYA28a further encodes an unusual zeaxanthin cluster (*crtE*–*idi*–*crtXYIBZ*) yielding fivefold more carotenoid [106] plus lipopolysaccharide biosynthesis and photolyase genes; together promoting UV resilience [107–110]. The SML is enriched in polysaccharides (Supplementary Fig. S4; Dunn’s test, adjusted *P* < .01), and NYA28a encodes diverse carbohydrate-active enzymes to utilize these: eight GH13 subfamilies, a PL5/PL14/PL38 cluster (likely targeting alginate), and a PL22 plus PQQ operon (likely targeting pectin). Together with the considerable sea-air and vertical transfer of carbohydrates—measured up to a kilometer via a tethered balloon in parallel to our microbial study [45]—these observations emphasize the connectivity between aquatic and atmospheric realms.

### Meteorological impact and environmental sourcing

**Figure 5:**
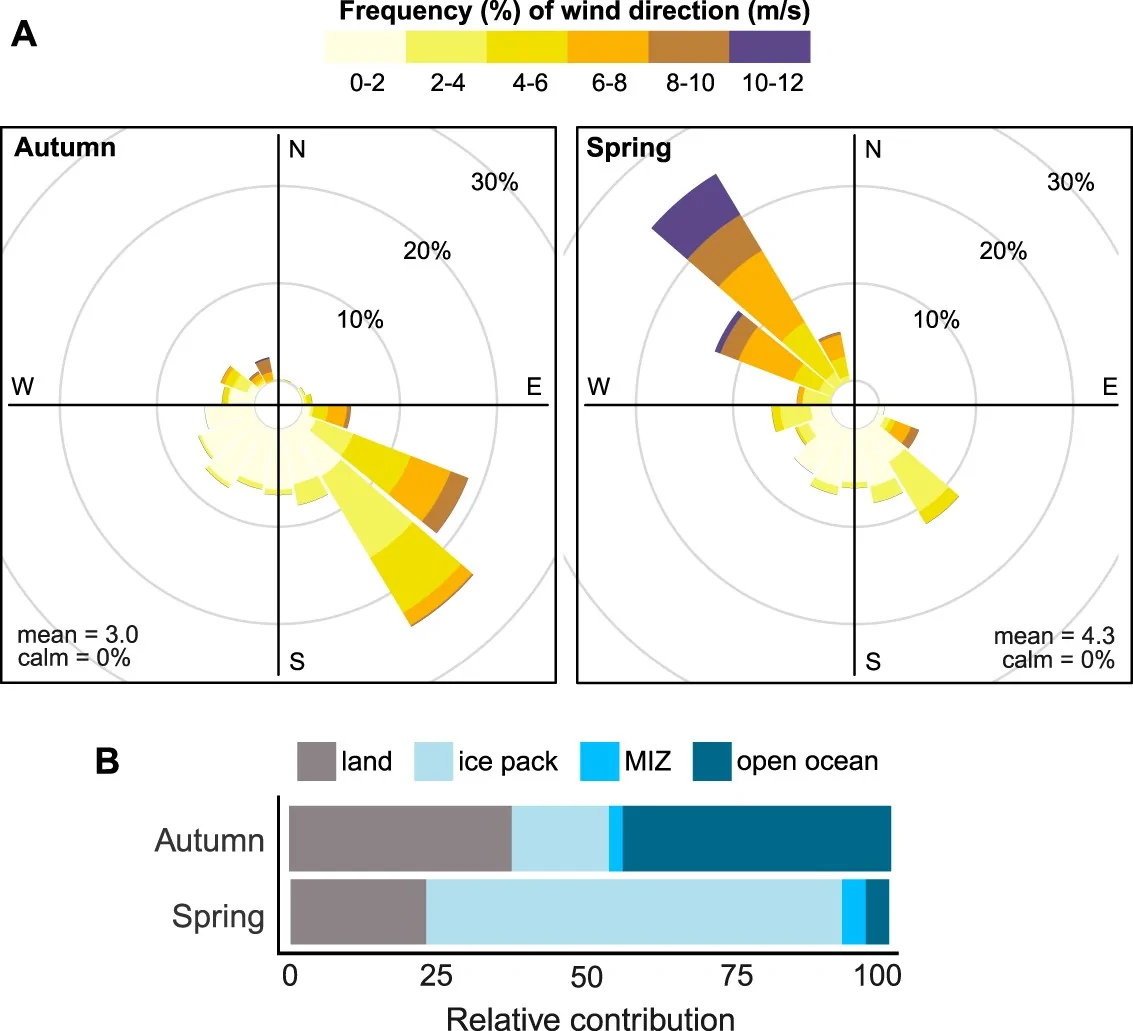
Seasonal environmental sourcing of aerosol microbiomes. (A) Frequency of wind directions and speed. B: Air mass back-trajectories showing the relative contribution of flow over land, the ice pack (>80% sea-ice cover), the marginal ice zone (MIZ; 15%–80% sea-ice cover), and the open ocean during the 48 h prior to arrival in Kongsfjorden. See Supplementary Fig. S5 for an animation.

The seasonally different aerosol microbiomes coincided with meteorological contrasts. Hourly meteorological data from the AWIPEV Observatory revealed significant seasonal differences of air temperature, air pressure, and wind speed (Supplementary Table S3, Wilcoxon rank-sum test, adjusted *P* < .001). Wind primarily came from southeast in autumn and northwest in spring (Fig. 5; Pearson’s chi-squared test with simulated p-value, χ^2^ = 242.87, *P* < .001, effect size = 0.48). Furthermore, back-trajectories revealed marked seasonal contrasts in air-mass histories (Supplementary Fig. S5), consistent with air flowing across distinct surfaces before reaching Kongsfjorden: open ocean in autumn and sea-ice in spring (Fig. 5; Wilcoxon rank-sum test, adjusted *P* < .001). Accordingly, predicting the origins of ASVs with reference to the ENVO database illustrated significantly higher marine aerosolization in autumn (Wilcoxon rank-sum test, adjusted *P* < .01).

**Figure 6:**
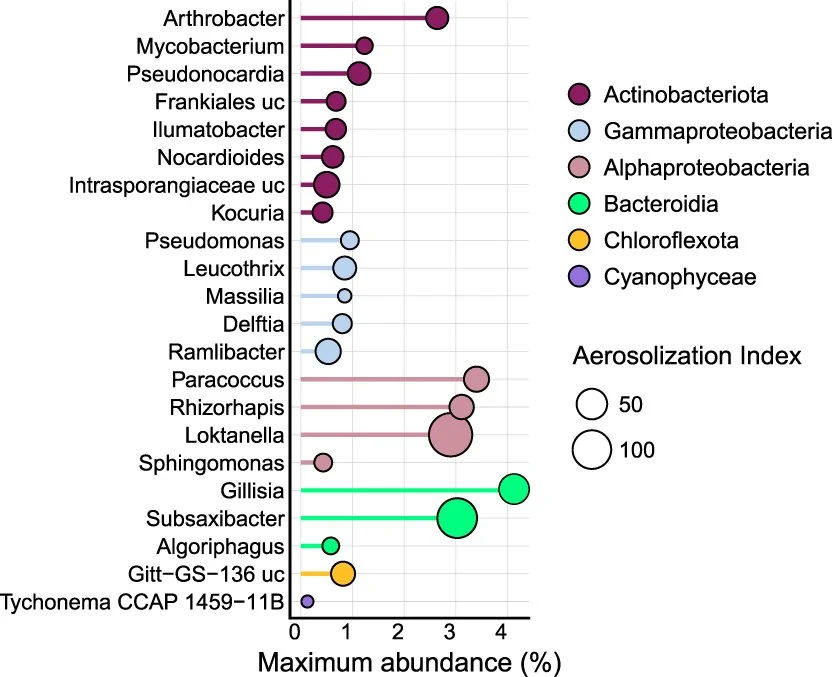
Snow in aerosol particle dynamics. Maximum abundance of bacterial genera co-detected in snow and aerosol particles. Aerosolization index and taxonomy are illustrated by dot size and color, respectively. uc: unclassified (only assigned to family level).

Our spring sampling occurred under heavy snow cover including fresh precipitation from two storms; promoting the aerosolization of snow bacteria (Fig. 6) such as *Arthrobacter* and *Ramlibacter* [111–113]. These dynamics resemble the cryosphere sourcing of spring air microbiomes in Greenland, driven by strong winds [114–116]. The aerosolization of *Tychonema* and *Massilia* reflects their known dispersal through snowstorms [116] and stress resistance in snow [117]. Detection of the lichen symbiont *Rhizorhapis* [118] and permafrost taxa like Gitt-136 [119] indicates additional dynamics during cryosphere-related aerosolization.

### Overarching ecosystem context

**Figure 7:**
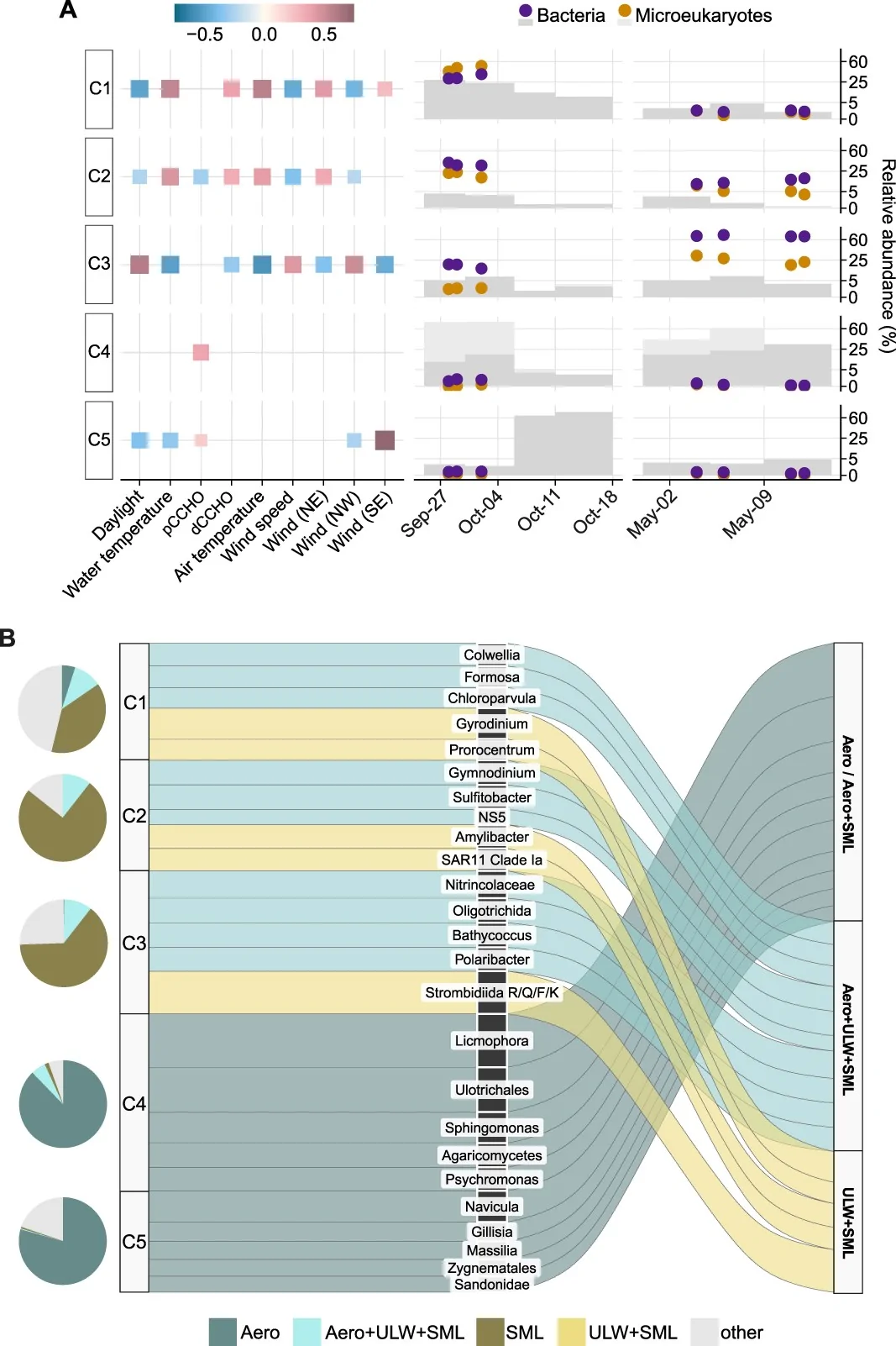
Ecosystem-scale connections between microbiomes, carbohydrates, and meteorology. (A) Delineation of five microbial clusters by their environmental correlations (left) and temporal dynamics (right). Colors in (A) illustrate the mean correlation across ASVs per cluster. Relative abundances for bacteria and eukaryotes are calculated separately per sample type. dCCHO: dissolved combined carbohydrates; pCCHO: particulate combined carbohydrates. (B) Contribution of environments to clusters (circles) and major corresponding genera (alluvial plot of square root-transformed relative abundances).

We return to an ecosystem-scale perspective by integrating bacterial and microeukaryotic dynamics with biochemical, physicochemical, and atmospheric variables. sPLS followed by hierarchical clustering of the correlation matrix resolved five clusters with distinct environmental, temporal, and taxonomic signatures (Fig. 7, Supplementary Fig. S6).

The autumn cluster C1, associated with dissolved combined carbohydrates, temperature and easterly winds, comprised typical late-bloom microbes, for instance *Gyrodinium* and *Prorocentrum* dinoflagellates [120]. Cluster C2 was comparably abundant in autumn and spring, but only contributed ~5% to aerosol microbiomes. C2 harbors *Amylibacter* and *Sulfitobacter* that degrade algal substrates like DMSP [34, 121]. Cluster C3 was most abundant in spring, represented by *Nitrincolaceae*, *Polaribacter* and *Bathycoccus* as commonly seen during early polar day around Svalbard [32, 96, 97]. The co-occurrence of different Strombidiida clades indicates diverse traits and niches of these ciliates; from autotrophic, heterotrophic to saprotrophic [122–124]. Clusters C4 and C5 comprise aerosolized microbes, with seasonal signatures: occurrence in early autumn and throughout spring (C4) compared to predominance in late autumn (C5; correlated with southeasterly winds). The presence of *Navicula*, *Ulotrichales*, and *Licmophora*, alongside positive correlations with particulate combined carbohydrates, illustrates a contribution of algal taxa and substrates to aerosol-specific clusters [125–128]. Two ASVs with matching genome-sequenced isolates are associated with cluster C5. For *Psychrobacter* asv515, the corresponding genome (NYA8) encodes a siderophore for iron acquisition and the machinery for assimilatory nitrate reduction to generate biomass from nitrate (Supplementary Table S2). NYA8 furthermore encodes a nitronate monooxygenase with 84% amino acid identity to a functionally characterized homologue from Antarctic *Psychrobacter* [129], suggesting a bipolar contribution to nitronate metabolism. For *Salinibacterium* asv4473, the corresponding genome (NYA9b) encodes a flexixanthin-related carotenoid and several other natural products (Supplementary Table S2), indicating substantial competitiveness.

### Biogeographic and biogeochemical implications

**Figure 8:**
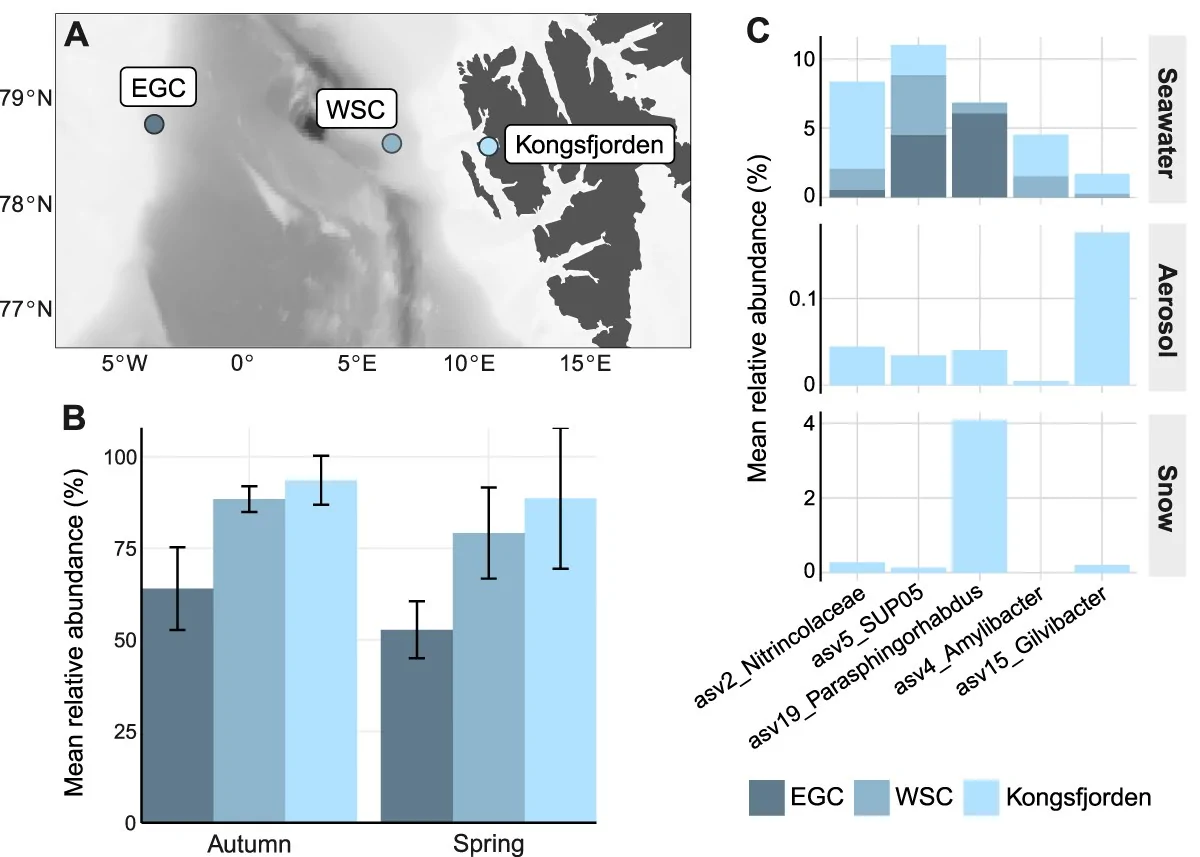
Implications for the wider Arctic. ASV distribution in Kongsfjorden seawater, aerosol particles, and snow in comparison with four-year microbiome records from the Fram Strait. (A) Location of Kongsfjorden and moored autonomous samplers in the Atlantic-influenced West Spitsbergen current (WSC) and the polar-influenced East Greenland current (EGC). (B) Combined relative abundance of Kongsfjorden “core taxa” (occurring in aerosol particles, ULW and SML at mean relative abundance >0.005) by season in comparison to WSC and EGC. **C:** Mean relative abundances of signature ASVs across sites and environments.

Multiple Kongsfjorden taxa are shared with microbiomes in the West Spitsbergen and East Greenland Currents (Fig. 8), illustrated through multiannual records from the FRAM Observatory [33, 34]*. Thioglobus* asv5—occurring in seawater, aerosol particles, and snow—is a “pan-Arctic resident” [34], highlighting sea–air connectivity from Svalbard fjords to the central Arctic Ocean. A genome-sequenced isolate from Svalbard waters with 100% rRNA gene identity [130] indicates that *Thioglobus* asv5 might perform mixotrophic carbon assimilation. *Nitrincolaceae* asv2 occurs in Kongsfjorden aerosols and the Atlantic-influenced West Spitsbergen Current, having 100% rRNA identity with isolates from the newly classified, ecologically diverse *Njordibacter* family [130]. *Parasphingorhabdus* asv19 prevailed in Kongsfjorden snow and the ice-covered East Greenland Current, highlighting the cryosphere contribution in spring including potential long-range advection. The corresponding genome (NYA22) encodes multiple biosynthetic gene clusters, including a zeaxanthin-related carotenoid for UV protection with homologs in glacier bacteria [107, 131]. Seven ribosomal peptides, a homoserine lactone, and a polyketide (Supplementary Table S2) suggest considerable potential for communication and competition. The presence of *narGHI* and *norB* genes indicate participation in both nitrification and denitrification.

## Conclusions

Our study illustrates how biology, meteorology, and physicochemistry shape the seasonal sea-air connectivity in Kongsfjorden, with broader implications for Arctic microbial ecology and glycobiology. We present three major conclusions:

1. Seasonally distinct “source microbiomes” across environmental gradients are a major driver of aerosol particle dynamics; likely promoted by genetic and phenotypic adaptations of seasonal communities [9, 15, 33, 34].
2. Aerosol particle microbiomes scale with meteorological conditions and air mass history, including the transfer of cryosphere microbes in spring. Potentially, aerosolized microbes represent “seeds” when precipitating from the atmosphere, especially when dispersed in dormant stages [22, 132].
3. Microbial similarities between Kongsfjorden, Fram Strait and the central Arctic Ocean indicates pan-Arctic patterns of aerosolization, linked to microbial connectivity via the transpolar drift, Atlantic water influx, and atmospheric dispersal.

## Supplementary Material

Supplementary_material_ycag212
