# Supplementary material for: Seasonal connectivity of microbes and carbohydrates between ocean, atmosphere, and cryosphere in Kongsfjorden (Svalbard, Arctic Ocean)
Source: ISME Commun. 2026 Jul 19;6(1):ycag212. doi: 10.1093/ismeco/ycag212 (PMC13518058; doi:10.1093/ismeco/ycag212)
Supplement: Supplementary file 1 [file supplementary_material_ycag212.zip › SI-info.docx]

**SUPPLEMENTARY DATA**

**Supplementary Figure S1: Seasonal microeukaryotic dynamics.** Non-metric multidimensional scaling of Bray-Curtis dissimilarities (A; stress value 0.06) and alpha-diversity metrics (B) of microeukaryotic communities in the surface microlayer (SML), the underlying seawater (ULW), and aerosol particles.

**Supplementary Figure S2:** **Fog water microbiomes.** Predominant prokaryotes in autumn fog water collected at the Gruvebadet Observatory, in comparison to the surface microlayer (SML) and the underlying seawater (ULW) in Kongsfjorden. PA: microbiome fraction >3 µm; FL: microbiome fraction 0.2 – 3 µm.

**Supplementary Figure S3:** **ASVs and corresponding bacterial isolates.** Average relative abundances of ASVs with 100% rRNA identity to genome-sequenced isolates by environment and season.

**Supplementary Figure S4: Carbohydrate concentrations across environments.** Values are µg L^-1^ for the surface microlayer (SML) and the underlying water (ULW), and ng m^-3^ for aerosol particles. dFCHO: dissolved free carbohydrates; dCCHO: dissolved combined carbohydrates; pCCHO: particulate combined carbohydrates.

**Supplementary Figure S5:** **Air mass histories.** Animated back-trajectories in autumn 2021 (left) and spring 2022 (right), determined hourly at three arrival altitudes (red 50 m, violet 250 m, pink 1000 m).

**Supplementary Figure S6: Ecosystem-scale connections between microbiomes, carbohydrates, and meteorology.** Sparse partial least square regression (sPLS) followed by hierarchical clustering on the correlation matrix identifies five clusters with distinct taxonomic and temporal casignatures. mean_T10_degC: air temperature (10 m height); mean_Pressure_hPa: atmospheric pressure; mean_Rha_% / mean_RHb_%: relative humidity; mean_FF10_m/s: wind speed (10 m height); DD_NE/NW/SE/SW/E/W/S: wind direction after converting decimal degrees into eight wind sectors; jday: Julian day; temp_C: water temperature; poreSize: filter pore size; dFCHO: dissolved free carbohydrates; dCCHO: dissolved combined carbohydrates; pCCHO: particulate combined carbohydrates; Fuc: fucose; Rha: rhamnose; Ara: arabinose; Gal: galactose; Glc: glucose; Xyl: xylose; Man: mannose; Fru: fructose; GalN: galactosamine; GlcN: glucosamine; MurAc: muramic acid; GalAc: galacturonic acid; GlcAc: glucuronic acid; ManAc: mannuronic acid.

**Supplementary Table S1:** Sample overview and metadata.

**Supplementary Table S2:** Seasonal microbial statistics, sPLS results, and genomic traits of bacterial isolates.

**Supplementary Table S3:** Carbohydrate concentrations and meteorological conditions, including statistical comparisons.
