# Supplementary material for: Seasonal connectivity of microbes and carbohydrates between ocean, atmosphere, and cryosphere in Kongsfjorden (Svalbard, Arctic Ocean)
Source: ISME Commun. 2026 Jul 19;6(1):ycag212. doi: 10.1093/ismeco/ycag212 (PMC13518058; doi:10.1093/ismeco/ycag212)
Supplement: Supplementary file 1 [file supplementary_material_ycag212.zip › FigureS1.pdf]

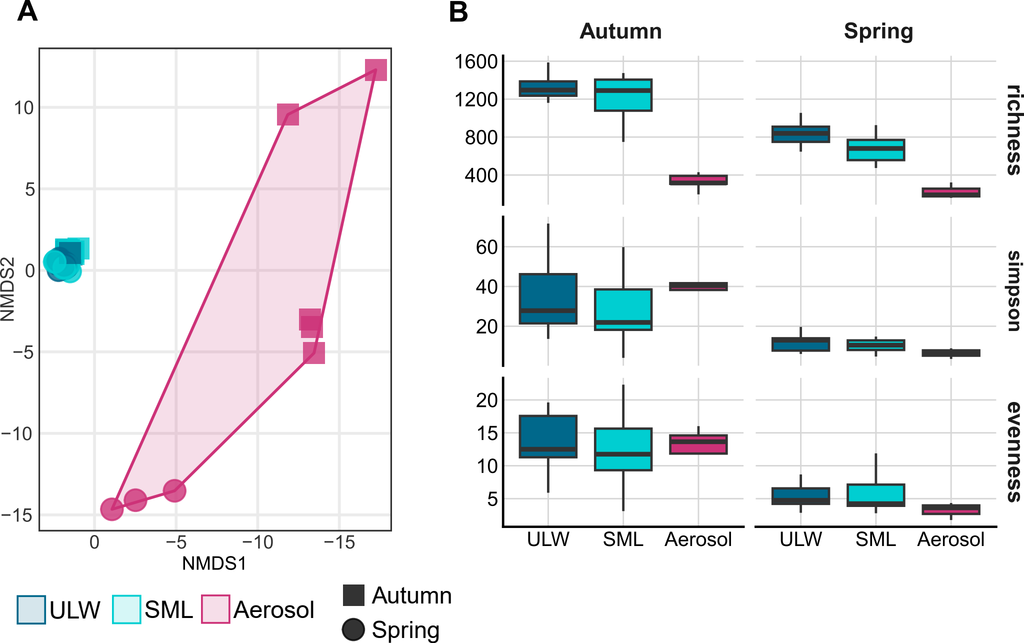

A
B
NMDS2
NMDS1
10
5
0
−5
−10
−15
0
−5
−10
−15
ULW
SML
Aerosol
Autumn
Spring
Autumn
Spring
1600
1200
800
400
richness
60
40
20
simpson
20
15
10
5
evenness
ULW
SML
Aerosol
ULW
SML
Aerosol
